# Adult-onset congenital cholesteatoma in the hypotympanum initially presenting as Bell’s palsy: A case report

**DOI:** 10.1097/MD.0000000000037511

**Published:** 2024-03-22

**Authors:** Pei-Shao Liao, Wei-Che Lan, Ching-Yuan Wang, Chia-Der Lin, Yu Aoh

**Affiliations:** a Department of Otolaryngology Head and Neck Surgery, China Medical University Hospital, Taichung, Taiwan; b School of Medicine, China Medical University, Taichung, Taiwan; c Department of Neurology, China Medical University Hospital, Taichung, Taiwan.

**Keywords:** Bell’s palsy, congenital cholesteatoma

## Abstract

**Introduction:**

Cholesteatoma is a rare disease characterized by the accumulation of keratinized squamous epithelial cells in the middle ear or mastoid cavity. Vertigo and facial palsy, which are rare complications, may indicate erosion into the semicircular canals or the fallopian canal.

**Patient concerns:**

A 40-year-old woman presented to our clinic with progressive right-sided hearing loss over 5 years (primary concern). Approximately 10 years ago, the patient had developed acute right-sided facial weakness with no additional symptoms. A neurologist at another hospital had diagnosed her condition as Bell’s palsy and treated it accordingly.

**Diagnosis:**

Adult-onset congenital cholesteatoma in the hypotympanum.

**Intervention:**

Combined endoscopic and microscopic removal of the cholesteatoma.

**Outcomes:**

Physical examination revealed slight improvement in right-sided peripheral facial palsy.

**Lesson:**

Routine eardrum examination is recommended for patients presenting with isolated peripheral facial palsy. If necessary, a patient should be referred to an otologist for further evaluation and treatment.

## 1. Introduction

Cholesteatoma refers to the accumulation of keratinized squamous epithelial cells in the middle ear or mastoid cavity. It is a rare disease with an incidence of 3 to 6 per 100,000 persons; congenital cholesteatoma accounts for 4% to 24% of the cholesteatomas in children and 2% to 5% of all cholesteatomas.^[1]^ The mean age at presentation is 5 to 7 years.^[2,3]^ Congenital cholesteatoma may develop even in the absence of any major otologic history and without any symptoms. Hearing loss, usually conductive, is probably the most common symptom of congenital cholesteatoma.^[3,4]^ Vertigo and facial palsy are rare complications and may indicate erosion into the semicircular canals or the fallopian canal.^[4,5]^ Potsic et al^[2]^ reported that the anterosuperior and posterosuperior quadrants of the eardrum are involved in 77% and 22% of all cases of congenital cholesteatoma, respectively.

Herein, we report the case of a middle-aged woman who presented to our clinic with right-sided facial palsy and recurrent right otalgia for over 2 years. This case report was prepared following the CARE guidelines.

## 2. Case presentation

A 40-year-old woman without medical or family history presented to our clinic with a primary concern of progressive right-sided hearing loss over a period of 5 years. Approximately 10 years ago, the patient had developed acute right-sided facial weakness without any additional symptoms and was treated for Bell’s palsy by neurologist at another hospital. However, her right-sided facial palsy showed no improvements. The patient reported having progressive right-sided hearing loss and recurrent vertigo (of varying durations) over the last 5 years but denied otorrhea, otalgia, or a history of any head trauma or otologic disease (Table 1).

**Table 1:** Timeline for clinical presentation.

Timeline	Events
10 years ago	Acute right-sided facial weakness, treated for Bell’s palsy by neurologist
5 years ago	Right-sided hearing loss
This time	Visited for progressive right-sided hearing loss and recurrent vertigo

**Figure 1.**
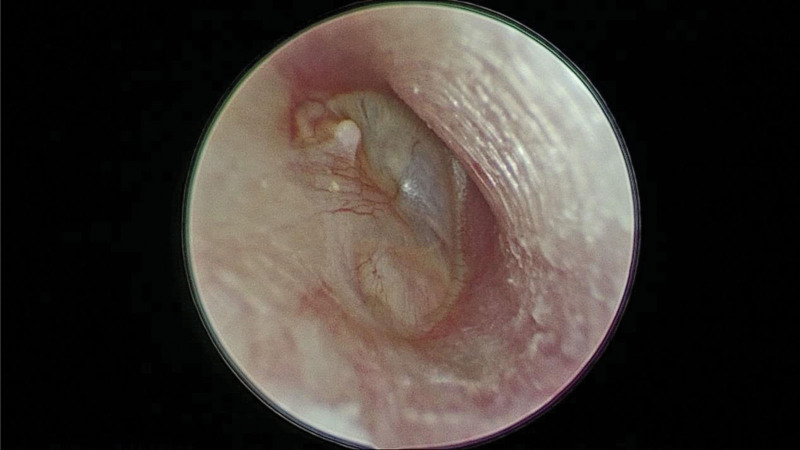
Whitish lesion noted over posteroinferior quadrant of intact eardrum.

Physical examination revealed a whitish lesion over the posteroinferior quadrant of the intact eardrum (Fig. 1). The results of additional head and neck examinations were unremarkable, except for right-sided facial palsy (House–Brackmann Facial Nerve Grading System, grade V).

Audiometric data, including pure-tone audiometry and speech audiometry, were collected.

**Figure 2.**
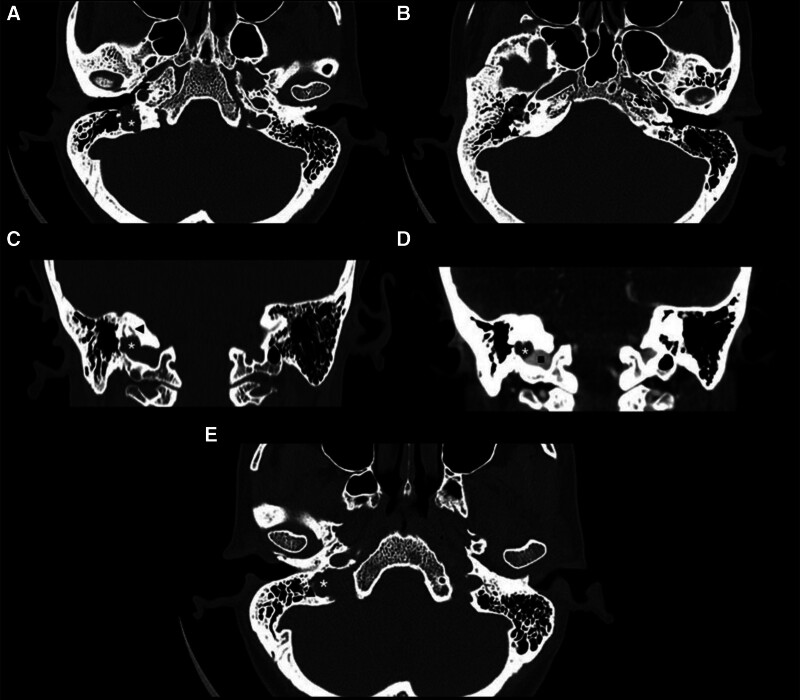
(A) Right middle ear lesion (asterisk) involving mastoid. (B) Right middle ear lesion involving lateral semicircular canal (black arrowhead). (C) Right middle ear lesion (asterisk) involving posterior semicircular canal (black arrowhead). (D) Nonenhanced lesion (asterisk) abutting right jugular bulb (black square) diverticulum. (E) Dehiscent mastoid segment of facial nerve (black arrowhead) abutting the lesion (asterisk).

The pure-tone average of the right ear was 53 dB HL, as measured using an air conduction test, and the air–bone gap was >30 dB HL. The speech reception threshold and speech discrimination score were 60 dB HL and 100% for the right ear, respectively. The audiometric results for the left ear were within normal ranges. Neither the ipsilateral nor the contralateral acoustic reflex was present in the right ear, whereas only the contralateral acoustic reflex was absent in the left ear. Evaluation of the motor nerve conduction velocity and blink reflex of the facial nerve revealed the presence of a lesion on the right facial nerve, below the level of the brainstem. High-resolution computed tomography of the mastoid behind the right ear showed a nonenhanced lesion involving the mastoid (Fig. 2A) and lateral (Fig. 2B) and posterior (Fig. 2C) semicircular canals, abutting the right jugular bulb diverticulum (Fig. 2D). A dehiscence was noted in the mastoid segment of the facial nerve (Fig. 2E); however, the tympanic segment of the facial nerve could not be traced because of the presence of the lesion in the right middle ear.

**Figure 3.**
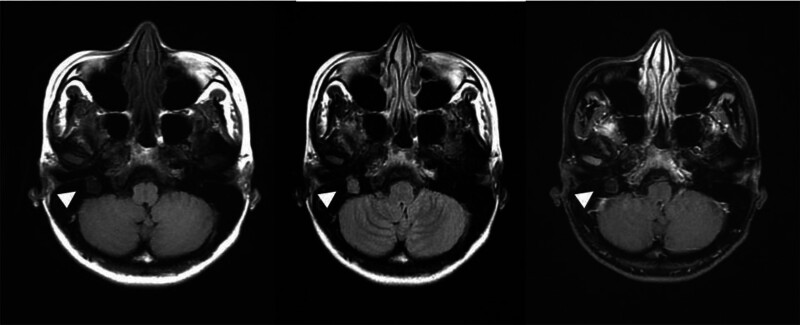
Lesion in right middle ear cavity: hypointense in T1-weighted scan (left), intermediate to hyperintense in T2-weighted scan (middle), and exhibited ring enhancement in postcontrast T1 scan (right).

Magnetic resonance imaging of the internal auditory canal (Fig. 3) revealed a 13-mm lobulated lesion in the right middle ear cavity, which was hypointense in a T1-weighted scan, was intermediate to hyperintense in a T2-weighted scan, and exhibited ring enhancement in a postcontrast T1 scan.

The patient was hospitalized for right transcanal endoscopic exploratory tympanotomy with canal wall-up mastoidectomy, and a retrofacial approach was adopted for the surgery, during which the facial nerve was monitored.

### 2.1. Endoscopic approach

**Figure 4.**
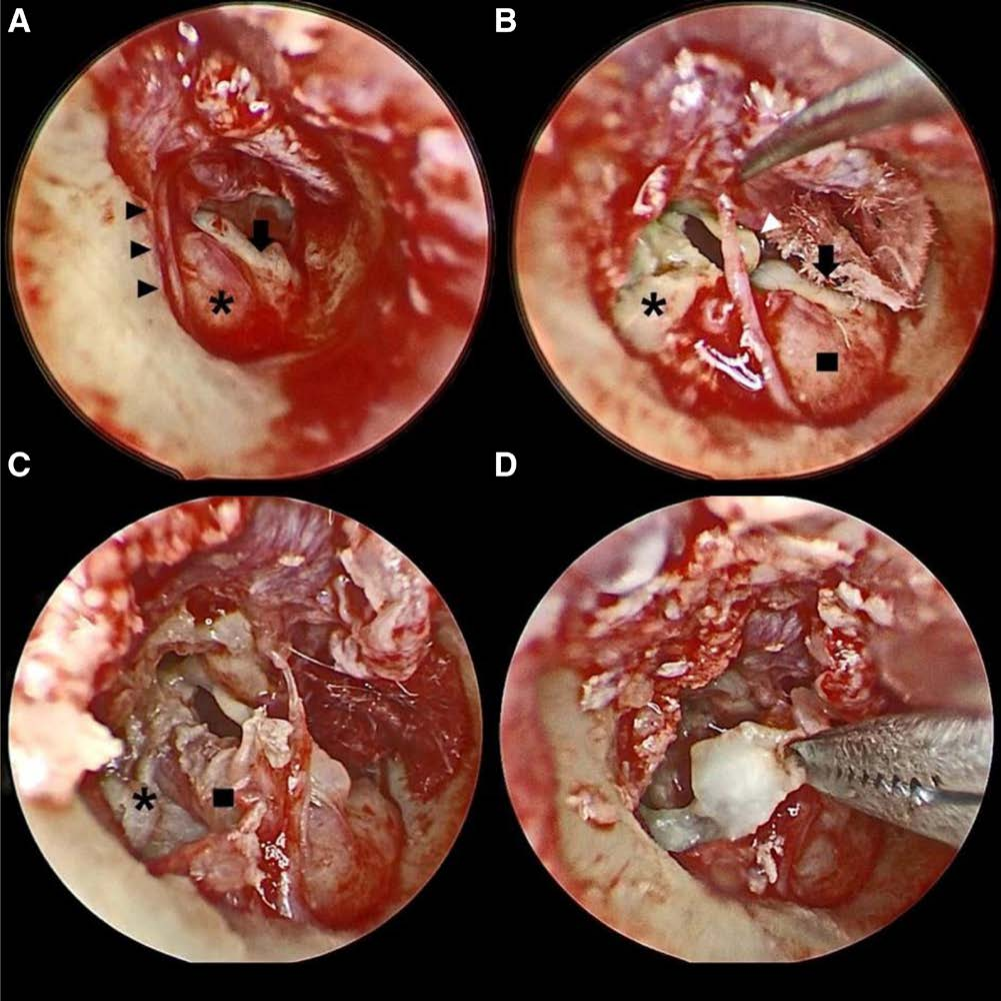
(A) Lesion (black arrow) noted behind facial nerve (asterisk); chorda tympani (black arrowhead). (B) After atticotomy, keratin mass (asterisk) observed in epitympanum; tensor tympani tendon (white arrowhead); unruptured cholesteatoma (black arrow) behind edematous facial nerve (black square). (C) Cholesteatoma extended into sinus tympani (asterisk); facial nerve (black square) inferiorly and laterally displaced and covering oval window region. (D) Removal of cholesteatoma from sinus tympani.

Exploratory tympanotomy was performed with the help of endoscopy, which was performed using a 3-mm endoscope. Tympanomeatal flap elevation revealed the edematous and lateralized tympanic segment of the facial nerve. The primary cholesteatoma lesion was noted in the mesotympanum, hypotympanum, and retromesotympanum. The intraoperative images are presented in Figure 4A to E. A keratin mass was noted in the epitympanum. After the partial removal of the cholesteatoma, we switched to microscopic surgery.

### 2.2. Microscopic approach

A postauricular incision (size, 8–10 mm) was made posterior to the postauricular sulcus. The alveolar fascia was harvested. A T-shaped incision was made through the soft tissues and the periosteum overlying the mastoid bone. Subsequently, the periosteum was elevated and retracted using self-retaining retractors to access the surgical area. Cortical mastoidectomy was performed, and the mastoid segment of the facial nerve was identified. The cholesteatoma was exposed through a retrofacial approach involving the removal of the bony plate separating the mesotympanum, hypotympanum, and mastoid cavity. Erosion of the posterior semicircular canal was noted in addition to jugular bulb dehiscence. The jugular bulb was in direct contact with the cholesteatoma in the hypotympanum. The cholesteatoma in the mesotympanum and hypotympanum was removed completely. Severe erosion of the lateral semicircular canal was observed. Further epitympanotomy was performed, followed by the complete removal of the keratin mass from the epitympanum. Bone chips and temporalis fascia were used to cover the dehiscent jugular bulb and the eroded posterior and lateral semicircular canals.

**Figure 5.**
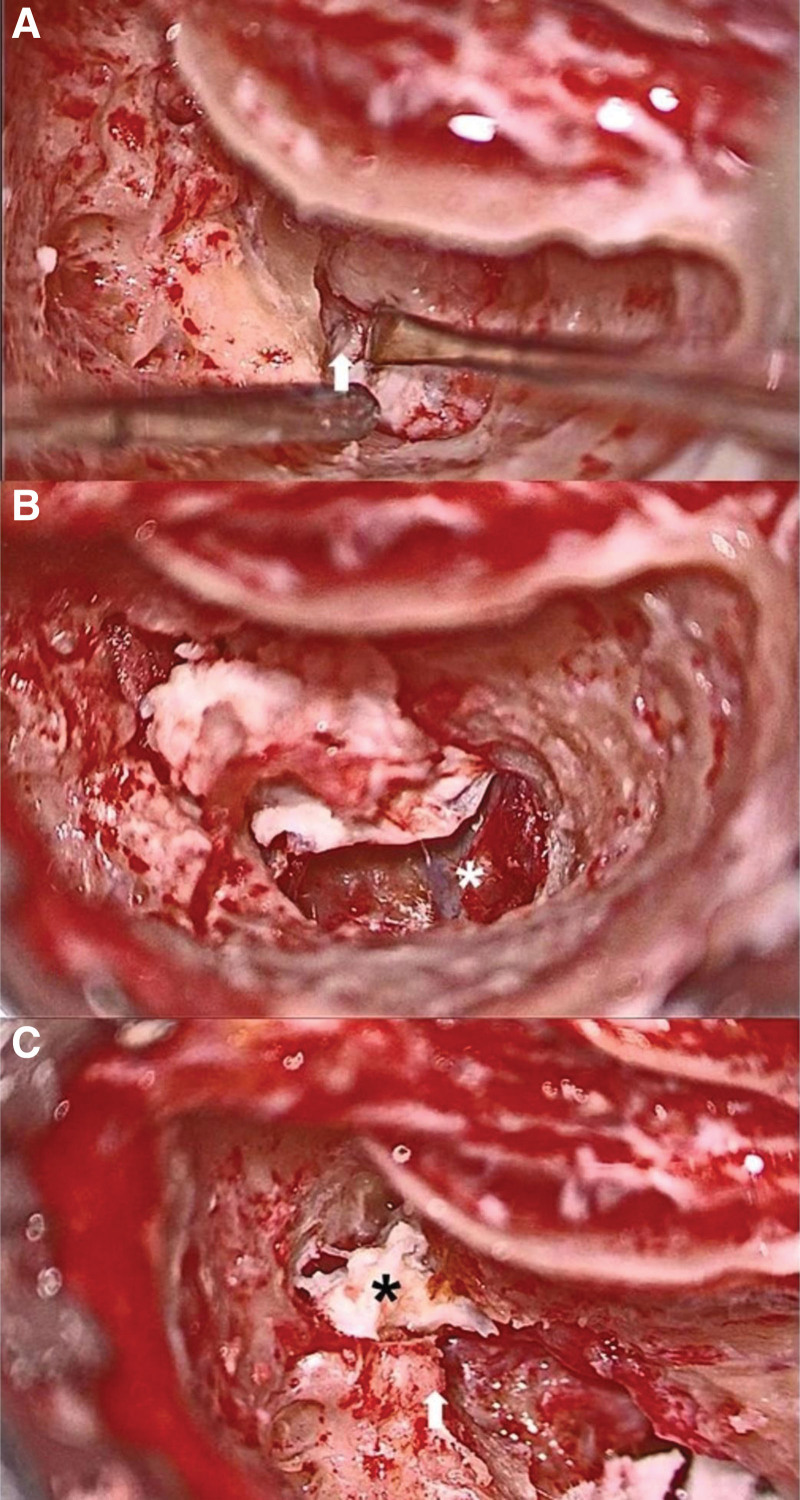
(A) Dehiscence of posterior semicircular canal (white arrow). (B) Dehiscence of jugular bulb (asterisk). (C) Presence of keratin mass (asterisk) in epitympanum and severe erosion of lateral semicircular canal (white arrow).

Intraoperatively, the chorda tympani was sacrificed because it was engulfed by the cholesteatoma. The facial nerve was found to be dehiscent over the tympanic segment and the upper part of the mastoid segment, with an aberrant route; the cholesteatoma pushed the facial nerve inferolaterally. The long process of the incus and the suprastructure of the stapes appeared to be absent. The oval window niche was covered by the aberrant and edematous tympanic segment of the facial nerve. Ossiculoplasty was not performed. The intraoperative images are presented in Figure 5A to C.

**Figure 6.**
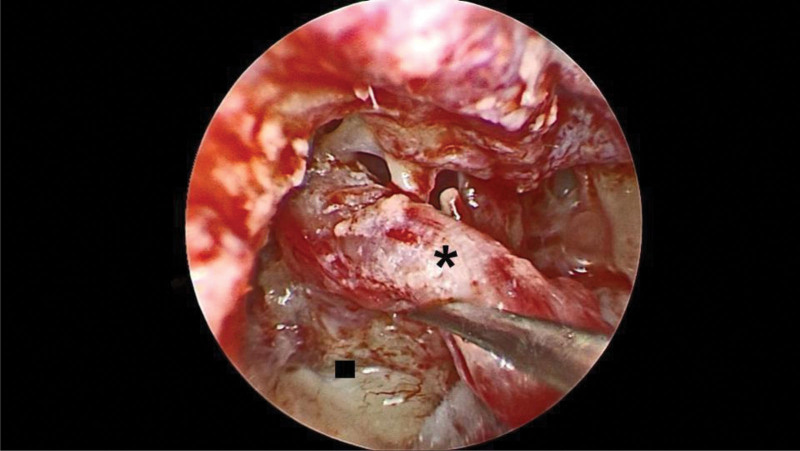
Clear sinus tympani and preserved facial nerve (asterisk).

At the end of surgery, we switched to transcanal endoscopy to reexamine hidden areas in the middle ear cavity, including the sinus tympani (Fig. 6). The middle ear and ear canal were packed with Gelfoam soaked in Tarivid ear solution, and the orifice of the external ear canal was secured with iodoform. The wound was closed layer by layer. The subcutaneous layer was sutured using a 4-0 Vicryl suture, and the skin incision was closed using a 4-0 Nylon suture. A Penrose drain was placed for fluid drainage. Finally, the postauricular wound was compressed and secured with loose gauze and elastic bandage. The patient exhibited good tolerance to the procedure.

Physical examination performed 1 month postoperatively revealed slight improvements in right-sided peripheral facial palsy; the House–Brackmann grade was III. Pure-tone audiometry performed 3 months postoperatively revealed that the air–bone gap was still >30 dB HL. The patient is currently under regular follow-up. The patient had given informed consent for this case report.

## 3. Discussion

Our patient had a history of right-sided peripheral facial palsy before the onset of hearing loss. Although the actual problem was congenital cholesteatoma, she was initially treated for Bell’s palsy; however, the patient had a poor response to the initial treatment. She did not consult any otorhinolaryngologist until the occurrence of progressive hearing loss and recurrent vertigo many years after the onset of facial palsy. Othman et al^[6]^ reported a case of congenital cholesteatoma mimicking Bell palsy in an adult patient. Therefore, routine eardrum examination is recommended for patients presenting with unilateral facial palsy to ensure early diagnosis. Potsic et al^[2]^ reported that 99% of all patients with congenital cholesteatoma have anterosuperior or posterosuperior quadrant involvement. However, our patient had a whitish lesion over the posteroinferior quadrant of the eardrum, although it is not a typical location for the development of congenital cholesteatoma. The unusual lesion location in our patient may explain the presentation of facial nerve palsy before the onset of hearing loss. Computed tomography of the mastoid behind her right ear revealed bone erosion, facial nerve dehiscence, and semicircular canal dehiscence, corresponding to the symptoms of facial palsy and recurrent vertigo. Cholesteatoma was suspected preoperatively. Magnetic resonance imaging of the internal auditory canal revealed that the lesion was hypointense in a T1-weighted scan and intermediate to hyperintense in a T2-weighted scan; these findings are consistent with the characteristics of cholesteatomas.^[7]^ Furthermore, intraoperative findings corroborated imaging data. Therefore, preoperative images must be carefully interpreted to ensure optimal outcomes.

## 4. Conclusion

Facial paralysis may be the only presentation in patients with middle ear cholesteatoma, which may be easily missed by physicians who are not otolaryngologists. Cholesteatoma should be regarded as a differential diagnosis for patients presenting with facial palsy. For these patients, the eardrum should be examined carefully. Audiometry and mastoid computed tomography should be performed if cholesteatoma is suspected. Preoperative image analysis is also crucial to avoid delay in diagnosis and treatment.

## Author contributions

**Data curation:** Wei-Che Lan.

**Investigation:** Wei-Che Lan.

**Project administration:** Wei-Che Lan, Yu Aoh.

**Supervision:** Wei-Che Lan, Ching-Yuan Wang, Chia-Der Lin, Yu Aoh.

**Writing – original draft:** Pei-Shao Liao.

**Writing – review & editing:** Wei-Che Lan, Yu Aoh.
